# 3D collagen fibrillar microstructure guides pancreatic cancer cell phenotype and serves as a critical design parameter for phenotypic models of EMT

**DOI:** 10.1371/journal.pone.0188870

**Published:** 2017-11-30

**Authors:** T. J. Puls, Xiaohong Tan, Catherine F. Whittington, Sherry L. Voytik-Harbin

**Affiliations:** 1 Weldon School of Biomedical Engineering, Purdue University, West Lafayette, Indiana, United States of America; 2 Department of Oncology, Eli Lilly and Company, Indianapolis, Indiana, United States of America; 3 Department of Basic Medical Sciences, Purdue University, West Lafayette, Indiana, United States of America; University of Alabama at Birmingham, UNITED STATES

## Abstract

Pancreatic cancer, one of the deadliest cancers, is characterized by high rates of metastasis and intense desmoplasia, both of which are associated with changes in fibrillar type I collagen composition and microstructure. Epithelial to mesenchymal transition (EMT), a critical step of metastasis, also involves a change in extracellular matrix (ECM) context as cells detach from basement membrane (BM) and engage interstitial matrix (IM). The objective of this work was to develop and apply an *in-vitro* three-dimensional (3D) tumor-ECM model to define how ECM composition and biophysical properties modulate pancreatic cancer EMT. Three established pancreatic ductal adenocarcinoma (PDAC) lines were embedded within 3D matrices prepared with type I collagen Oligomer (IM) at various fibril densities to control matrix stiffness or Oligomer and Matrigel combined at various ratios while maintaining constant matrix stiffness. Evaluation of cell morphology and protein expression at both the cellular- and population-levels revealed a spectrum of matrix-driven EMT phenotypes that were dependent on ECM composition and architecture as well as initial PDAC phenotype. In general, exposure to fibrillar IM was sufficient to drive EMT, with cells displaying spindle-shaped morphology and mesenchymal markers, and non-fibrillar BM promoted more epithelial behavior. When cultured within low density Oligomer, only a subpopulation of epithelial BxPC-3 cells displayed EMT while mesenchymal MiaPaCa-2 cells displayed more uniform spindle-shaped morphologies and mesenchymal marker expression. Interestingly, as IM fibril density increased, associated changes in spatial constraints and matrix stiffness resulted in all PDAC lines growing as tight clusters; however mesenchymal marker expression was maintained. Collectively, the comparison of these results to other in-vitro tumor models highlights the role of IM fibril microstructure in guiding EMT heterogeneity and showcases the potential of standardized 3D matrices such as Oligomer to serve as robust platforms for mechanistic study of metastasis and creation of predictive drug screening models.

## Introduction

Pancreatic ductal adenocarcinoma (PDAC) is one of the deadliest cancers with an estimated 5-year survival rate of around 5% [1]. PDAC is characterized by an intense stromal reaction, known as desmoplasia, where overactive cancer associated fibroblasts deposit excessive extracellular matrix (ECM), the bulk of which is fibrillar type I collagen [2,3]. It is widely thought that this stromal remodeling and dysregulation of cell-ECM homeostasis serves to promote cancer progression, including metastasis and drug resistance [2,4]. However, recent evidence suggests that desmoplasia may paradoxically play an important protective role, where resulting changes in ECM composition and architecture restrict rather than promote tumor growth and invasion [5]. Clearly, tumor-stromal ECM interactions play a critical role in PDAC pathophysiology; however, advanced *in-vitro* and *in-vivo* models are needed to achieve a more complete mechanistic understanding [5–7]. This knowledge gap, which exists for not only PDAC, but most solid tumors, precludes development of novel targeted therapies as well as identification of better predictors of patient therapeutic response. Since patients generally die from metastatic disease and PDAC has such a high metastasis rate, better understanding of how stromal ECM guides tumor phenotype and behavior is paramount to improving clinical outcomes [8–10].

**Fig 1:**
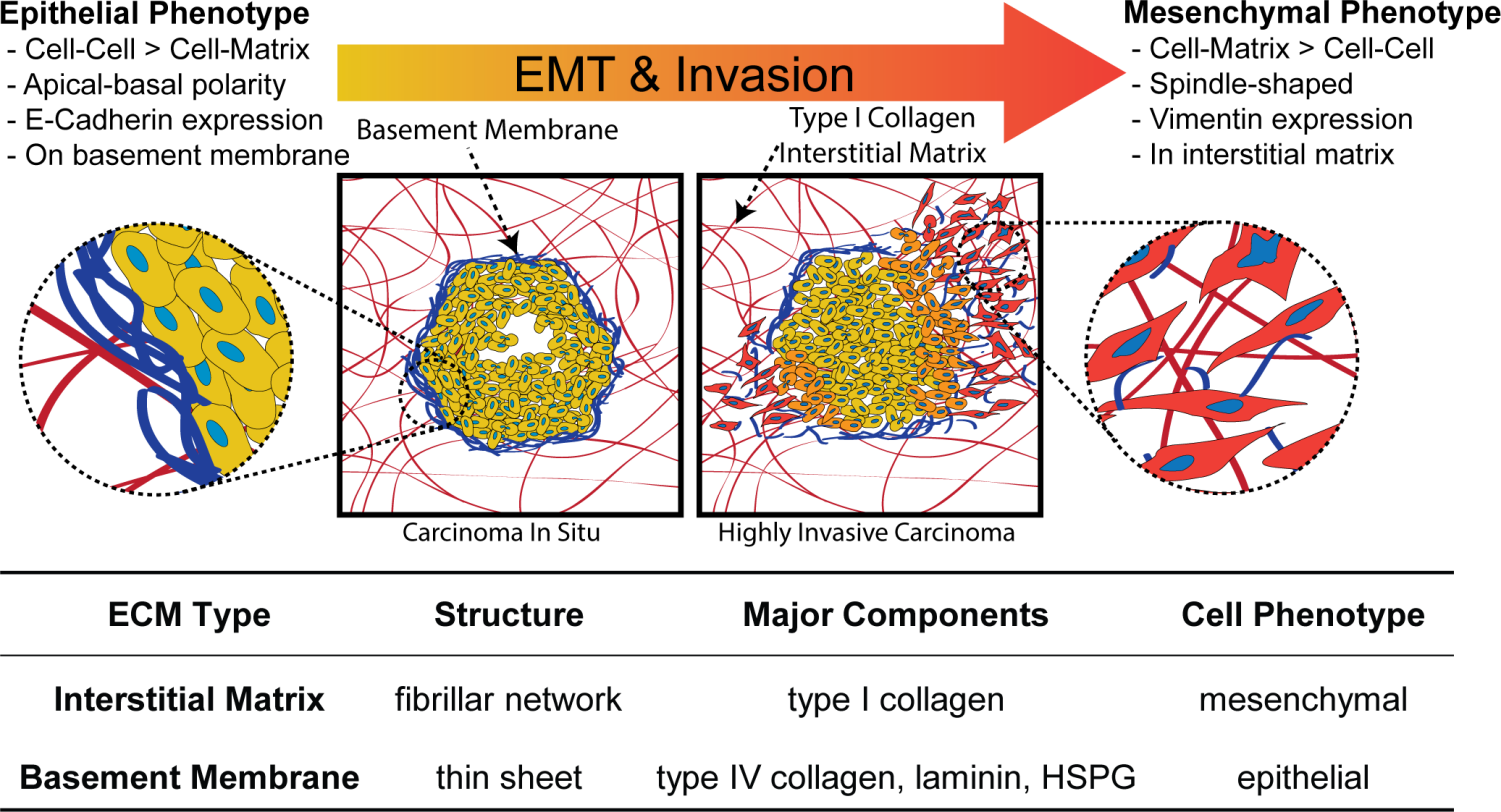
Overview of EMT and associated tumor stromal ECM interactions. Schematic shows key characteristics and progression of EMT, as epithelial cancer cells lose contact with basement membrane and interface with the surrounding interstitial matrix which is composed primarily of fibrillar type I collagen. Association with the stromal interstitial matrix correlates with transition from an epithelial to a mesenchymal phenotype. HSPG = heparan sulfate proteoglycan.

ECM associated with PDAC, as well as normal tissues, is represented by two distinct types, namely basement membrane (BM) and interstitial matrix (IM). BM, composed primarily of laminin, non-fibrillar type IV collagen, and heparan sulfate proteoglycan, forms a thin sheet-like structure which supports and polarizes epithelial cell layers, separating them from the underlying interstitial tissue compartment. In contrast, the predominant component of IM is fibrillar type I collagen, within which individual mesenchymal cells (e.g., fibroblasts) reside. It is noteworthy that a hallmark of tumor metastasis is epithelial to mesenchymal transition (EMT), where epithelial cancer cells lose polarity and cell-cell associations while gaining a more mesenchymal and invasive phenotype. Fig 1 highlights salient features of tumor EMT, drawing attention to the altered stromal ECM context encountered by tumor cells as they increasingly interact with surrounding IM [9]. This marked difference in ECM context is often overlooked in conventional EMT schematics where IM is often excluded and intracellular events are emphasized [9,11].

Although it is evident that EMT is marked by dynamic tumor cell-ECM interactions, where cells may engage both BM and IM components, many experimental *in-vitro* models lack rigorous definition of relevant ECM molecular and physical properties [12,13]. In fact, tumor EMT research has traditionally focused on soluble factor (e.g., TGF-β1) induction and intercellular signaling cascades [14,15]. However, since recent work suggest that matrix composition and physical properties (e.g. microstructure and viscoelastic properties) are involved in driving EMT, ideal tumor models should recreate the 3D fibrillar IM microstructure and geometry of cell-cell and cell-matrix associations to support physiologically relevant cell phenotypes, as well as enable mechanistic study through matrix tunability [2,16,17]. Unfortunately, many models fail to replicate these critical features of 3D tumor-ECM interactions and give rise to contradictory results. For example, breast cancer cells cultured on-top of Matrigel- or type I collagen-coated polyacrylamide (PA) gels showed that increased PA concentration (stiffness) enhanced EMT [18,19], while breast cancer and glioblastoma spheroids embedded within 3D fibrillar type I collagen matrices showed decreased mesenchymal behavior and invasiveness with increased collagen concentration (stiffness) [20–22]. These examples showcase how different geometries of cell-matrix interactions and ECM microstructures can yield conflicting results. Clearly, the ability to recreate ECM architecture and physical properties experienced by cancer cells *in vivo* will contribute to a more complete understanding of EMT and invasion.

Over the last several years, our laboratory has worked to develop new tools and methods for mechanistic study of fibrillar type I collagen self-assembly and the impact of resultant matrix physical properties on cell phenotype, function, and tissue morphogenesis. As part of this effort, we identified and standardized (ASTM F3089-14) a new soluble collagen subdomain (Oligomer) that retains natural, mature intermolecular crosslinks as well as the uncommon capacity for suprafibrillar self-assembly [23,24]. Oligomer displays rapid self-assembly (polymerization) *in vitro* and *in vivo*, forming highly-interconnected, D-banded collagen-fibril networks, which are similar to those found in tissues *in vivo*. This collagen formulation supports systematic modulation of the physical and biological properties of 3D IM microenvironments beyond what can be achieved with conventional collagen monomer formulations [23]. The tunable and robust matrices formed with Oligomer have proven useful in a variety of *in-vitro* applications including mesenchymal stem cell differentiation, vascular network formation, glioblastoma migration, and tissue engineering [22,25–27]. Type I oligomeric collagen (Oligomer) offers a promising alternative to materials traditionally used to mimic the IM in 3D cancer models.

The goal of the present work was to develop and apply an *in-vitro* 3D tumor-ECM model relevant to PDAC to further define the importance of ECM composition and physical properties in guiding EMT. Three well-characterized PDAC lines representing points along the EMT phenotypic spectrum were embedded within 3D self-assembled ECM microenvironments of defined composition and physical properties and changes in their phenotype evaluated. For this work, Oligomer was used to recreate the IM, and Matrigel, a reconstituted BM isolated from Engelbreth-Holm-Swarm (EHS) mouse sarcomas (a tumor rich in basement membrane) [28] was used to mimic the BM. Matrigel is routinely used to approximate a BM-like environment since it provides a complex mixture of macromolecules including laminin, type IV collagen, and enactin. However, limitations associated with Matrigel include: 1) poorly defined molecular composition, 2) lot-to-lot variability (protein concentration and molecular composition), and 3) inability to recapitulate the specific nature of the biochemical and biophysical environment associated with all tumors (lacks type I collagen and *in-vivo* cross-linking) [28,29]. Overall, our results revealed a spectrum of matrix-driven phenotypes that were dependent on ECM composition, fibril architecture, and initial cell phenotype. In general, non-fibrillar BM (Matrigel) promoted epithelial behaviors such as clustered cell growth and E-cadherin expression. In low density Oligomer, the exposure to fibrillar IM promoted EMT of a subpopulation of epithelial BxPC-3 cells, while mesenchymal MiaPaca-2 cells more uniformly displayed mesenchymal characteristics. Interestingly, for all PDAC lines, increasing IM fibril density, which represents increasing desmoplasia, resulted in confined clustered growth due to the increased spatial constraints and matrix stiffness. Comparison of ours results to those from other *in-vitro* models highlight the importance of ECM composition, microstructure, and mechanical properties in 3D *in-vitro* cancer models and establish Oligomer as a powerful tool in cancer research for mechanistic study of tumor-ECM interactions.

## Materials and methods

### Cell culture

BxPC-3, Panc-1, and MiaPaCa-2 cell lines were all obtained from American Type Culture Collection (ATCC, Manassas, VA) and maintained per manufacturer guidelines. BxPC-3 cells were grown in RPMI-1640 (Life Technologies, Grand Island, NY) and Panc-1 and MiaPaCa-2 cells were grown in high glucose DMEM (Hyclone, Logan, UT), all supplemented with 10% heat inactivated fetal bovine serum (HI FBS; Life Technologies), 100 U/mL penicillin, and 100 μg/mL streptomycin (Sigma Aldrich, St. Louis, MO). Growth medium of MiaPaCa-2 cells was additionally supplemented with 2.5% horse serum (Sigma Aldrich). Cells were maintained in a humidified environment of 5% CO_2_ in air at 37°C, passaged at 70–80% confluency, and used in experiments at passage numbers between 6 and 14.

### Creating *in-vitro* 3D tumor-ECM models

Type I oligomeric collagen was extracted from the dermis of market weight pigs (Beutler Meat Processing, Lafayette, IN) using acid solubilization as previously described [23]. Pig hides for this extraction process were obtained from a commercial meat-processing source according to Purdue University Animal Care and Use Committee (PACUC) guidelines, though specific approval was not needed. Extracted collagen was lyophilized for storage, dissolved in 0.01 N hydrochloric acid (HCl) for use, and standardized according to ASTM International Standard F3089-14 [30]. Briefly, Oligomer formulations were standardized based on molecular composition and polymerization capacity which is defined by the relationship between shear storage modulus (G’, Pa) of the polymerized matrix as a function of Oligomer concentration. Growth Factor Reduced Matrigel (Corning Life Sciences, Tewksbury, MA) was stored at -20°C and thawed at 4°C or on ice before use. Since Matrigel is known to have high batch-to-batch variability [28,31], all drug dosing experiments were performed with the same lot.

*In-vitro* 3D tumor-ECM models were created by encapsulating tumor cells within various reconstituted matrix compositions at 2x10^5^ cells/mL. Matrigel was used undiluted, while Oligomer was diluted with 0.01 N HCl to desired concentrations and neutralized (pH = 7.4) with 10X phosphate-buffered saline (PBS) and 0.1 N sodium hydroxide as previously described [25]. To create matrices with different Oligomer:Matrigel ratios, neutralized Oligomer solution (0.9 mg/mL) and Matrigel solutions were admixed at the following ratios—100:0, 75:25, 50:50, 25:75, 0:100 (volume:volume). To determine the effect of IM physical properties on EMT, Oligomer-only matrices were prepared at concentrations of 0.9, 1.5, 2.1 mg/mL which corresponded to shear storage modulus (matrix stiffness) values of approximately 100-, 500- and 1000 Pa. We have previously established that a positive correlation exists between Oligomer concentration, fibril density, and matrix stiffness [23,25,32]. 3D constructs were created by aliquoting matrix-cell suspensions (100 μL, 2x10^5^ cells/mL) into a 96 well plate followed by incubation at 37°C for 20–30 minutes to induce matrix polymerization or self-assembly. Immediately following self-assembly, the appropriate cell culture medium was added and constructs were cultured for four days with medium changes every other day.

### Viscoelastic testing

Viscoelastic properties of matrices were determined using oscillatory shear mode on an AR2000 rheometer (TA Instruments, New Castle, DE) as previously described [23]. Samples were polymerized on the rheometer stage for 30 min followed by a shear-strain sweep from 0.1% to 4% strain at 1 Hz. The shear storage modulus (G’) at 1% strain was used as a measure of matrix stiffness. Each sample was tested in triplicate (n = 3).

### Staining and imaging

Tumor-ECM constructs were fixed in 3% paraformaldehyde (Mallinckrodt, Derbyshire, UK) and permeabilized using 0.1% Triton X-100 (Sigma Aldrich). To visualize cell morphology, F-actin was stained with Alexa Fluor 488 phalloidin (Life Technologies). For immunostaining, constructs were blocked with 1% bovine serum albumin (Jackson ImmunoResearch, West Grove, PA), followed by overnight incubation at 4°C with goat anti-rabbit primary antibodies for vimentin and E-cadherin (D21H3 and 24E10, Cell Signaling Technologies, Danvers, MA). After rinsing with 1X PBS, constructs were incubated overnight at 4°C with anti-rabbit conjugated Alexa Fluor 488 secondary antibody (A12379, Life Technologies) followed by nuclear counterstaining with Draq5 (Life Technologies).

Images were collected using laser scanning confocal microscopy on an Olympus IX81 inverted microscope with an Olympus Fluoview FV1000 system (Olympus, Tokyo, Japan). Image stacks of 150–200 μm thickness with a 5 μm step size were obtained using a 20X air or 60X water objective, and z-projections were created using Imaris software (Bitplane, Concord, MA). Confocal reflection microscopy was used to visualize the collagen-fibril microstructure [33].

### Western blotting

Western blots were used to determine EMT protein expression on a population level for BxPC-3 and MiaPaca-2 in 2D and within constructs of varied Oligomer stiffness and Oligomer:Matrigel ratios. Cell lysates from 2D culture were obtained directly from cell culture flasks at 70–80% confluency using chilled 1X RIPA buffer (Millipore, Bedford, Massachusetts) containing 0.2% halt phosphatase and protease inhibitor cocktail (Thermo Fisher Scientific, Waltham, MA), and 2% phenylmethanesulfonyl fluoride solution (Sigma-Aldrich; 2%). Lysates from 3D culture were obtained by snap freezing constructs after four days of culture, grinding them into a powder, and dissolving in lysis buffer. All samples were kept on ice with periodic vortexing for one hour for 2D samples and three hours for 3D samples. Total protein concentration for all samples was determined using a BCA protein analysis kit (Pierce, Rockford, Illinois). Samples containing 30 μg of protein were loaded onto a 4–20% Tris-HCl pre-cast gels (Bio-Rad, Hercules, CA) and transferred onto Trans-Blot Turbo Midi Nitrocellulose membranes (Bio-Rad). After blocking in SEA BLOCK Blocking Buffer (Thermo Fisher Scientific) overnight at 4° C, the membranes were incubated with mouse antibodies against E-cadherin (Cell Signaling Technology, 1:1000), fibronectin (BD Biosciences, San Jose, CA; 1:1000), and vimentin (BD Biosciences; 1:1000) overnight at 4° C. Mouse antibody against glyceraldehyde 3-phosphate dehydrogenase (GAPDH; Meridian Life Science, Memphis, TN; 1:1000) was used as a loading control. Membranes were then washed in 1X PBS with 0.05% Tween-20 (Sigma-Aldrich) and incubated for two hours at room temperature with horseradish-peroxidase–conjugated IRDye 800CW anti-mouse secondary antibody (LI-COR Biosciences, Lincoln, NE; 1:10000). After multiple washes with the PBS/Tween solution, bands were visualized using Odyssey CLx Infrared Imaging System (LI-COR).

### Determining gemcitabine sensitivity

PDAC cell sensitivity to gemcitabine (Santa Cruz Biotechnology, Dallas, TX) was determined by dosing with a 10-point drug dilution (1:5 dilution starting at 200 μM) and calculating IC50 values from data obtained with Alamar Blue metabolic indicator (Invitrogen, Frederick, MD). Cells were seeded in 96-well plates either on 2D tissue culture plastic at 4x10^3^ cells/well or within different 3D matrix formulations at 2x10^5^ cells/mL (100 μL or 2x10^4^ cells/well). After 24 hours, culture medium was replaced with medium containing gemcitabine dilutions, 20 μM staurosporine (positive kill control; Santa Cruz Biotechnology), or 1% DMSO (negative control; Sigma Aldrich). Treatments were replenished after 48 hours. Each treatment was performed in triplicate, and all experiments were repeated at least 3 times (N≥ 3; n = 3).

After 72 hours of treatment, fresh medium containing 10% Alamar Blue solution and 1% FBS was added to each well, and well-plates were incubated for an additional 8 hours. Fluorescence intensity of medium was measured spectrofluorometrically using 530 nm/590 nm excitation/emission on a SpectraMax M5 Microplate Reader (Molecular Devices, Sunnyvale, CA). Raw intensity values were normalized to the positive and negative controls with the following equation: % *Cell Viablity* = (I_*n*_ − I_*STS*_)/(I_*DMSO*_ − I_*STS*_) × 100%. *I*_*n*_ represents the intensity value of the *nth* dilution. *I*_*STS*_ and *I*_*DMSO*_ represent intensities recorded from positive kill control and negative control, respectively. GraphPad Prism (GraphPad Software Inc., San Diego, CA) was used to fit a four-parameter logistic curve. The automatic robust outlier detection algorithm within Prism was used to detect and exclude outliers from the final fit. Reported values represent relative IC50, defined as the halfway point between the bottom and top plateaus of each curve.

### S-phase fraction determination

Total cell number and the fraction of cells undergoing S-phase were determined using Click-iT Edu (Life Technologies) followed by quantitative image analysis using Imaris (Bitplane). Briefly, after 3 days of culture, tumor-ECM construct medium was refreshed with medium containing 10 μM 5-ethynyl-2’-deoxyuridine (Edu) and cultured for another 24 hours. Constructs were then fixed with 3% paraformaldehyde (Mallinckrodt), permeabilized using 0.1% Triton X-100 (Sigma Aldrich), and incubated with Click-iT reaction cocktail prepared following manufacturer’s instructions. Constructs were subsequently counterstained with Draq5 and z-stack images of 50 μm thickness were collected using laser scanning confocal microscopy on an Olympus IX81 inverted microscope with an Olympus Fluoview FV1000 system (Olympus) and a 20X air objective. The Imaris spot detection algorithm was used to independently detect nuclei stained with Click-iT Edu and Draq5 for quantification of the number of cells undergoing S-phase and total cell number, respectively. S-phase fraction was then calculated by dividing the number of S-phase cells by the total number of cells. Images (2 per well) from two separate experiments (N = 2) performed in triplicate (n = 3) for each matrix stiffness were used for final calculations and statistics.

### Statistical analysis

Statistical analyses were performed using SAS (Statistical Analysis System; SAS Institute Inc., Cary, NC). For gemcitabine sensitivity, a two-factor ANOVA was used to compare stromal ECM microenvironment and cell type. Main effects were compared using Tukey-corrected pairwise comparisons for one factor while holding the other factor constant. A one-factor ANOVA with Tukey-corrected pairwise comparisons was used to analyze viscoelastic testing data. S-phase fraction data was analyzed with a three-factor ANOVA which included experiment number and replicate as factors in order to test their significance and not falsely inflate the degrees of freedom in the model. In all cases, differences were considered statistically significant when p<0.05.

## Results

### Established PDAC lines cultured in 2D represent phenotypes along the EMT spectrum

**Fig 2:**
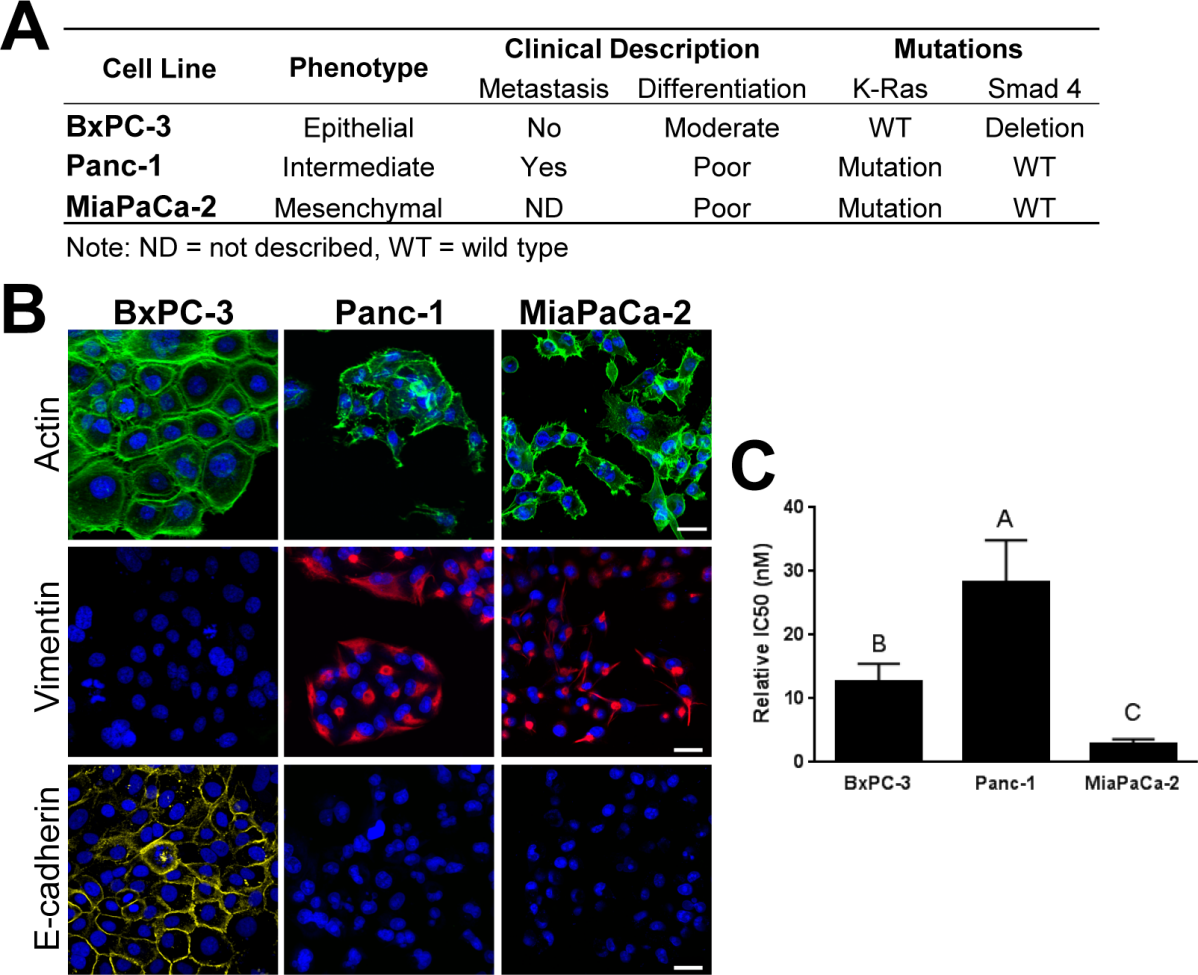
PDAC lines differ in EMT phenotype and gemcitabine resistance when cultured on 2D plastic. (A) Table summarizing pancreatic cell line phenotype, patient derivation, and relevant mutations [36]. (B) BxPC-3, Panc-1,and MiaPaCa-2 cells were cultured on 2D plastic (4x10^3^ cells/well) for 4 days, stained for actin (green), vimentin (red), E-cadherin (yellow), and nuclei (blue), and imaged using confocal microscopy. Images represent z-stack projections (20 μm thickness; scale bar = 30 μm). (C) Gemcitabine IC50 values (mean ± SD) were determined from 10-point dose response curves for cell lines cultured on 2D plastic. Letters indicate statistically different groups (p<0.05, n = 5).

Throughout this work, three established PDAC lines with distinct EMT phenotypes were used (BxPC-3 –epithelial, Panc-1 –intermediate, and MiaPaCa-2 –mesenchymal) to evaluate ECM-guided EMT. Literature based characteristics of these PDAC lines are summarized in Fig 2A. To validate each cell line’s initial phenotype, cell morphology and expression patterns of EMT marker proteins, E-cadherin and vimentin [8,34], were determined following 2D culture. Here, we defined epithelial phenotype as cells growing in tight clusters with prominent E-cadherin expression, high cell-cell interactions, and primarily cortical actin [35]. On the other hand, mesenchymal phenotype was characterized by individual cells with spindle-shaped morphology, prominent actin projections, high cell-ECM interactions, and pronounced vimentin expression [35].

Consistent with previously published work [37,38], BxPC-3 cells displayed an epithelial phenotype in 2D culture, growing as cell clusters with prominent E-cadherin expression, while MiaPaCa-2 cells were mesenchymal, displaying the typical spindle-morphology with vimentin expression (Fig 2B). Panc-1 cells exhibited an intermediate phenotype, growing as clusters with some actin projections and expressing vimentin but not E-cadherin (Fig 2B). In addition to phenotype, gemcitabine sensitivity was measured with relative IC50 values calculated from 10-point dose response curves, showing that in 2D, Panc-1 cells were the most resistant to gemcitabine, followed by BxPC-3, and then MiaPaCa-2 (Fig 2C). These results were consistent with previously published studies where similar experimental conditions and assay methods were employed (S1 Table).

### At matched stiffness, Oligomer induces EMT and Matrigel induces MET

The effect of various ECM ligands and soluble factors on tumor cell plasticity and EMT phenotype is routinely studied for cells cultured on 2D surfaces [39–42]. However, less is known regarding how cells sense and respond to 3D IM and BM environments with defined biochemical and biophysical attributes [21,43,44]. Here, PDAC lines were cultured within 3D type I collagen-fibril matrices prepared with Oligomer and tumor BM-like microenvironments prepared with Matrigel. Since Oligomer and Matrigel represent different ECM compositions and microstructures, an Oligomer concentration was chosen to yield matrix stiffness values that matched undiluted Matrigel (G’ = 100 Pa), avoiding stiffness as a possible confounding variable.

**Fig 3:**
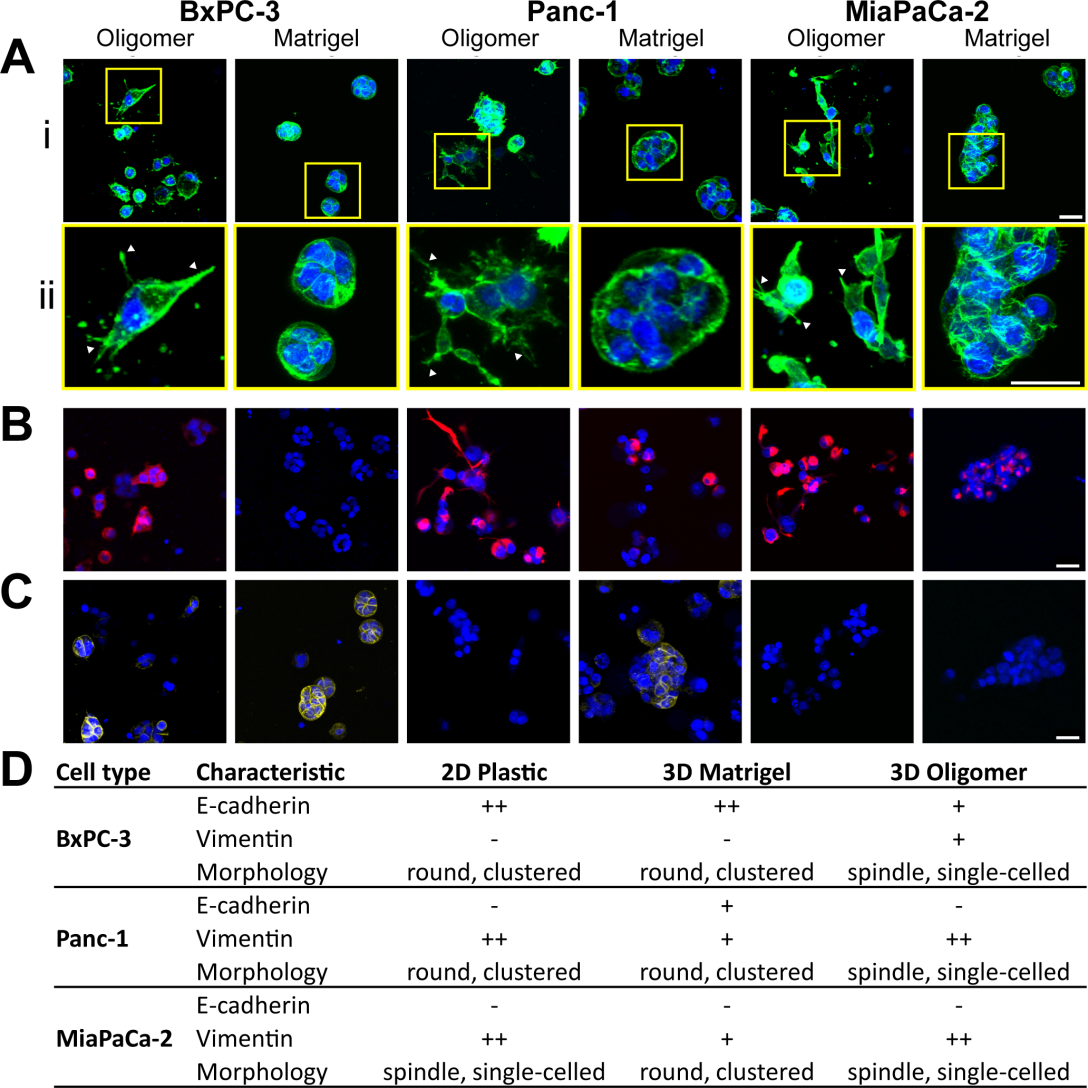
Stromal ECM drives pancreatic cancer cell morphology and phenotype. BxPC-3, Panc-1, and MiaPaCa-2 cells were cultured (2x10^5^ cells/mL) for 4 days within Oligomer (0.9 mg/mL; 100 Pa) and Matrigel (100 Pa). Constructs were stained for actin (A; green), vimentin (B; red), E-cadherin (C; yellow), and nuclei (blue) and imaged using confocal microscopy. Images represent z-stack projections (100 μm thickness; scale bar = 30 μm). Yellow boxes represent 3X digitally zoomed sections (ii) and arrowheads note prominent actin protrusions. (D) Table summarizing protein expression and morphological observations from panels A-C.

As summarized in Fig 3, the fibrillar type I collagen matrices formed by Oligomer (100 Pa) promoted various degrees of EMT and mesenchymal behavior, while Matrigel induced more MET and epithelial behavior. More specifically, when grown within Oligomer (100 Pa), all PDAC lines showed decreased cell-cell associations and more pronounced spindle-shaped morphology with prominent actin projections. A subset of BxPC-3 cells shifted to expressing both vimentin and E-cadherin in Oligomer as detected by immunostaining, while both Panc-1 and MiaPaCa-2 cells only expressed vimentin. In contrast, all PDAC lines cultured within Matrigel grew as tight clusters with high nuclear to cytoplasmic ratios and cortical actin (Fig 3). Additionally, within Matrigel, Panc-1 cells shifted to expressing both E-cadherin and vimentin, while BxPC-3 and MiaPaCa-2 cells expressed E-cadherin only and vimentin only, respectively. Collectively, these results document that specific ECM ligands, and their associated microstructures, are critical determinants of PDAC phenotype and behavior, especially as it relates to EMT and MET.

### Dose response analysis reveals matrix-dependent gemcitabine sensitivity of PDAC lines

**Fig 4:**
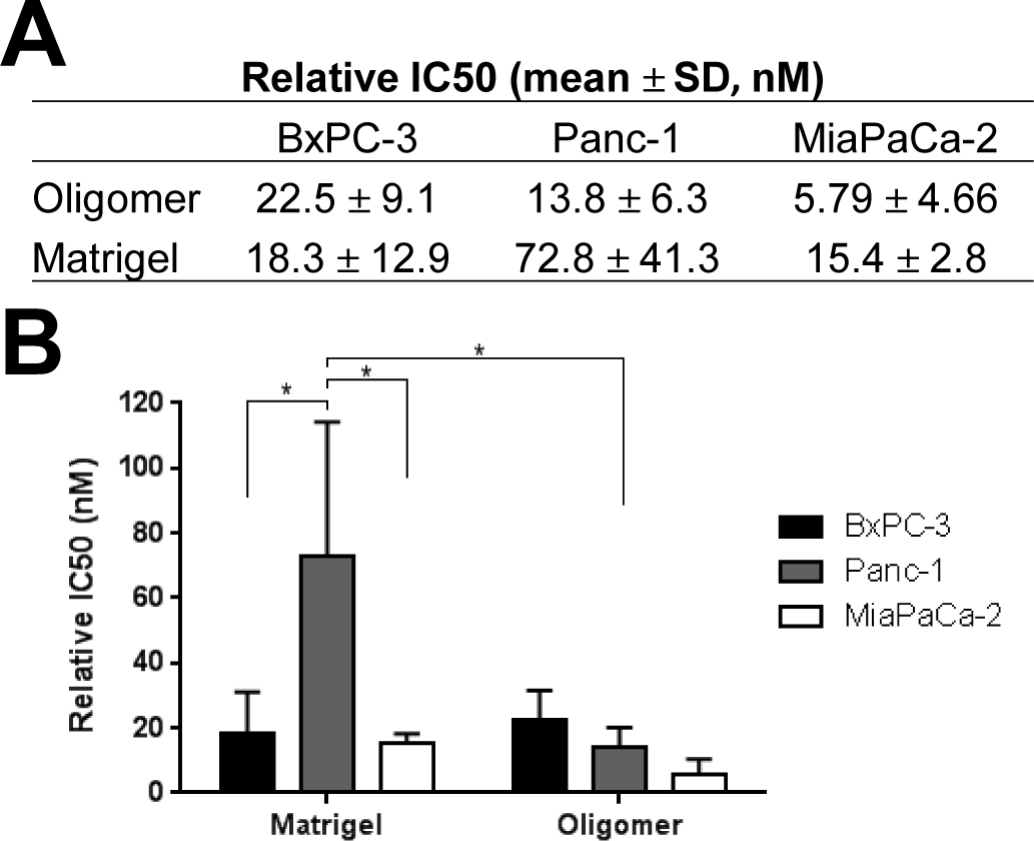
Sensitivity of PDAC lines to gemcitabine depends on 3D matrix type. (A) Table and (B) graph summarizing gemcitabine IC50 values (mean ± SD) for PDAC lines cultured (2x10^5^ cells/mL) for 4 days within Oligomer (0.9 mg/mL, 100 Pa) or Matrigel (100 Pa). Asterisk (*) indicates statistically different groups (p<0.05, N = 3–4, n = 3).

In high throughput 2D drug screening, IC50 is commonly used as a measure of drug sensitivity. However, fewer studies report IC50 values for 3D culture models because adapting drug dosing protocols and analyses to 3D formats tends to be more difficult, time consuming, and resource intensive [45,46]. To demonstrate that our 3D tumor-ECM model is amenable to medium and high throughput drug screening, the relative IC50 values of gemcitabine were determined for each PDAC line within 3D Oligomer and Matrigel, prepared with matched matrix stiffness. As shown in Fig 4, gemcitabine sensitivity of PDAC lines was matrix dependent. When cultured within Oligomer (100 Pa), PDAC lines showed statistically similar (p>0.05) IC50 values, ranging from about 6 to 23 nM. In contrast, when cultured within Matrigel, Panc-1 cells displayed a significantly (p<0.05) higher IC50 value of 72.8±41.3 nM compared to BxPC-3 and MiaPaCa-2 cells which measured 18.3±12.9 nM and 15.4±2.8 nM, respectively. Further, IC50 values for BxPC-3 and MiaPaCa-2 cells in Matrigel were statistically similar (p>0.05) to those obtained for Oligomer (100 Pa), while Panc-1 cells’ IC50 was significantly lower (p<0.05) in Oligomer compared to Matrigel. It is worth noting that Panc-1 cells showed greater resistance to gemcitabine compared to the other PDAC lines when cultured within Matrigel (Fig 4) and on plastic (Fig 2C), but not within Oligomer. Collectively, these results emphasize that the tumor microenvironment, including ECM composition and microstructure, is a critical determinant of PDAC drug sensitivity.

### Varying stromal IM to BM ratio guides PDAC phenotype and EMT

As tumor cells become exposed to fibrillar type I collagen, whether deposited by stromal cells or encountered at the tumor-tissue interface, cells may simultaneously interact with both IM and BM components, thus engaging different integrin receptors [47,48]. To define how such dynamic cell-ECM signaling affects PDAC phenotype and EMT, PDAC cells were cultured within matrices prepared with different ratios of Oligomer and Matrigel, representing IM and BM, respectively. These experiments were conducted with only two PDAC lines, BxPC-3 and MiaPaCa-2, representing epithelial and mesenchymal phenotypes.

**Fig 5:**
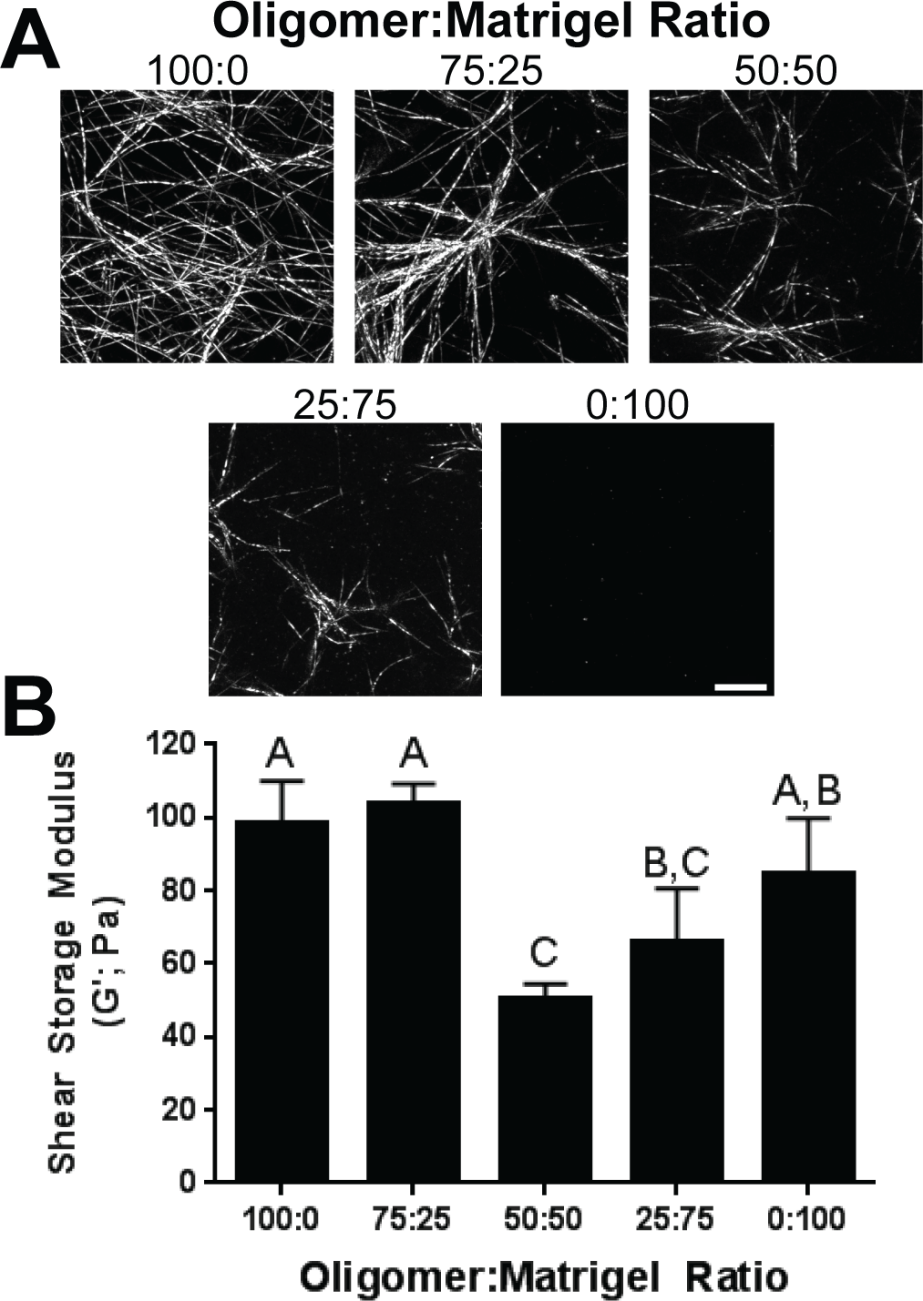
Oligomer:Matrigel ratio affects matrix microstructure and stiffness. (A) Images represent z-stack projections (10 μm thickness; scale bar = 10 μm) from confocal reflection microscopy of matrices prepared with varying Oligomer:Matrigel ratios. (B) Matrix stiffness values are given as shear storage modulus (G’; mean ± SD). Letters indicate statistically different groups (p < 0.05, n = 3).

Matrix microstructure and stiffness were defined to determine how varying Oligomer:Matrigel (IM:BM) ratio altered matrix self-assembly and physical properties (Fig 5). As visualized by confocal reflectance microscopy, matrix architecture varied from the highly- branched fibrillar network for Oligomer (100:0) to no visible fibril microstructure in Matrigel (0:100), emphasizing differences in self-assembly capacity and matrix physical properties. Fibril density and length appeared to decrease with decreasing Oligomer:Matrigel ratio with 100:0 and 75:25 displaying dense branched fibril networks and 50:50 and 25:75 exhibiting fewer and shorter fibrils. Observed differences in microstructure were consistent with measured alterations in matrix mechanical properties. More specifically, matrix stiffness (G’) values for 100:0 and 75:25 were statistically similar (p>0.05) while 50:50 and 25:75 were significantly softer (p< 0.05). As expected for this experiment, 0:100 (Matrigel) and 100:0 (Oligomer) stiffness values were statistically similar (p>0.05), since Oligomer concentration was chosen to match the stiffness of undiluted Matrigel.

**Fig 6:**
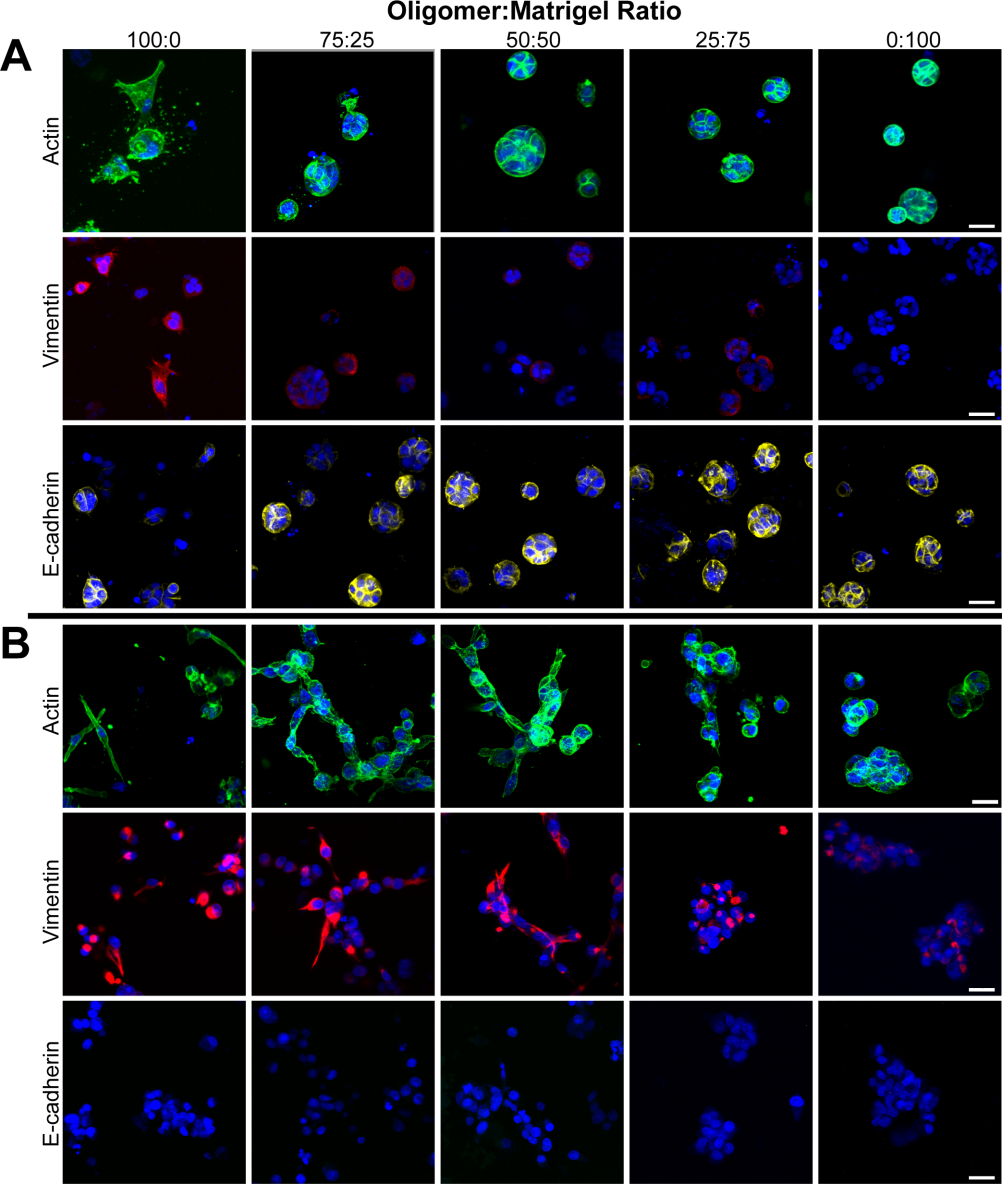
Oligomer:Matrigel ratio modulates EMT phenotype. (A) BxPC-3 and (B) MiaPaCa-2 cells were cultured (2x10^5^ cells/mL) within 3D matrices prepared with various Oligomer:Matrigel ratios for 4 days. Constructs were stained for actin (green), vimentin (red), E-cadherin (yellow), and nuclei (blue) and imaged using confocal microscopy. Images represent z-stack projections (100 μm thickness; scale bar = 30 μm).

**Fig 7:**
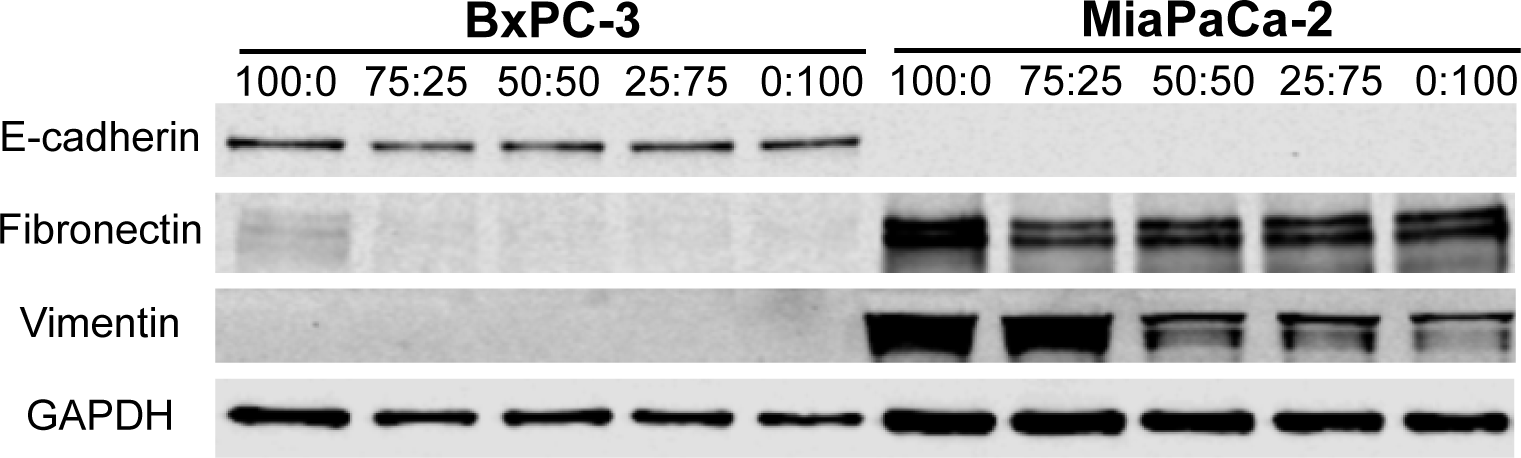
Oligomer:Matrigel ratio effects on population level EMT protein expression is dependent upon initial PDAC phenotype. Western blots showing EMT protein expression for BxPC-3 and MiaPaCa-2 (2x10^5^ cells/mL) cultured for 4 days within 3D matrices prepared at various Oligomer:Matrigel ratios.

Analysis of PDAC cell morphology and protein expression as a function of Oligomer:Matrigel ratio further supported the supposition that Oligomer induced EMT and Matrigel induced MET to an extent that was dependent upon initial PDAC cell phenotype. As the Oligomer:Matrigel ratio increased, an increased number of BxPC-3 cells transitioned from an epithelial morphology to smaller cell clusters or individual cells displaying prominent cytoplasmic projections (Fig 6A). In addition, these subpopulations showed an apparent increase in vimentin expression and decreased E-cadherin expression as the Oligomer:Matrigel ratio increased. Interesting these differences in maker protein expression were not observed when measured at a population level via western blots which showed no change in E-cadherin expression and no detectable vimentin expression with changing ratios (Fig 7). Modest fibronectin expression was observed in BxPC-3 cells cultured in 100:0, but expression decreased to undetectable levels once Matrigel was introduced into the construct.

MiaPaCa-2 cells, on the other hand, showed more drastic changes in morphology (Fig 6B) and population-level protein expression (Fig 7) with varying Oligomer:Matrigel ratio, while immunostaining revealed only modest changes in EMT protein expression patterns (Fig 6B). Within the lowest Oligomer:Matrigel ratios (0:100 and 25:75), MiaPaCa-2 cells were tightly packed with little to no actin projections. As Oligomer content increased, cells became progressively more singular and spindle-shaped, with cells in 75:25 aligning into networks resembling the invasive front of a tumor. Immunostained MiaPaCa-2 cells did not express detectable E-cadherin in any matrix composition. While vimentin expression was observed in all matrices, it appeared to be reduced within 0:100 and 25:75 and shifted from uniformly distributed throughout the cytoplasm to localized around cell nuclei. Analysis by western blot confirmed immunostaining results with Oligomer:Matrigel ratio showing having no effect on MiaPaCa-2 E-cadherin expression and vimentin expression decreasing with Matrigel content (Fig 7). A similar decreasing expression trend was observed for fibronectin with highest expression levels occurring in MiaPaCa-2 cells cultured within 100:0. Altogether, these results suggest that i) a combination of IM and BM interactions plays an important role in guiding tumor cell plasticity and EMT, and ii) ECM type and microstructure differentially regulate cell phenotype depending on the cells’ initial EMT status.

### Increasing collagen-fibril density (matrix stiffness) controls mesenchymal behavior and suggests matrix dependent correlation between S-phase fraction and drug sensitivity

**Fig 8:**
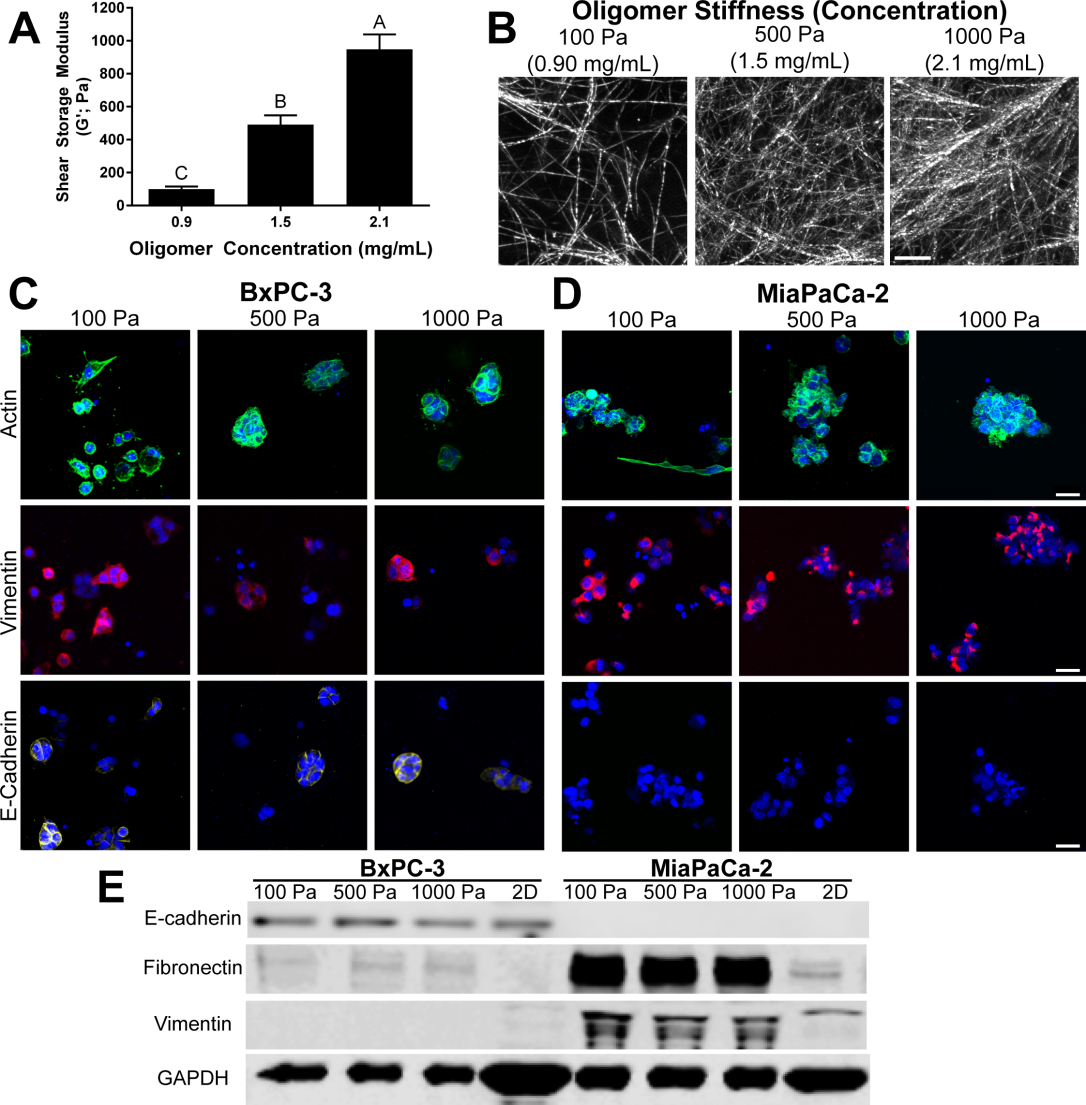
Stromal interstitial matrix stiffness alters PDAC cell phenotype. (A) Matrix stiffness values of matrices prepared at Oligomer concentrations of 0.9, 1.5, 2.1 mg/mL are given as shear storage modulus (G’; mean ± SD) with letters indicating statistically different groups (p < 0.05, n = 3). (B) Images represent z-stack projections of confocal reflection microscopy (10 μm thickness, scale bar = 10 μm) of matrices (C) BxPC-3 and (D) MiaPaCa-2 cells were cultured (2x10^5^ cells/mL) within these three matrices for 4 days. Constructs were stained for actin (green), vimentin (red), E-cadherin (yellow), and nuclei (blue) and imaged using confocal microscopy. Images represent z-stack projections (100 μm thickness; scale bar = 30 μm). (E) Western blot measurement of EMT protein expression for BxPC-3 and MiaPaCa-2 cultured for 4 days within various Oligomer matrices and on 2D tissue culture plastic.

Pancreatic cancer is known for significant desmoplasia, characterized by over-active stellate cells and fibroblasts which increase fibrillar type I collagen deposition [3,49]. However, the precise role played by desmoplasia and type I collagen in tumor-stromal ECM interactions and tumor progression remains uncertain [5,50,51]. In order to better define the role of IM physical properties in regulating EMT and drug sensitivity, matrix stiffness was varied by altering the Oligomer concentration or fibril density as seen in Fig 8A and 8B. The positive correlation between oligomer concentration, fibril density, and matrix stiffness has been established previously [23,25,32]. PDAC lines were cultured within Oligomer matrices prepared with stiffness values of 100, 500, and 1000 Pa and analyzed for changes in phenotype, S-phase fraction, and gemcitabine sensitivity. The S-phase fraction was quantified since gemcitabine causes checkpoint arrest and cell death by incorporating into DNA during S-phase [52].

When cultured in low stiffness (100 Pa) Oligomer, a significant subpopulation of BxPC-3 and nearly all MiaPaCa-2 cells took on a spindle-shaped, mesenchymal morphology. However, as Oligomer density and stiffness increased, both cell types grew as clustered aggregates, displaying more epithelial-like behavior (Fig 8C and 8D). Western blot data showed no appreciable changes in EMT marker protein expression as a function of Oligomer stiffness (Fig 8C–8E). BxPC-3 expressed E-cadherin in the majority of cells in immunestained samples and also showed prominent bands for E-cadherin. On the other hand, while immunostaining showed vimentin expression in some BxPC-3 cells, expression was not detectable in western blots. Conversely, MiaPaCa-2 showed vimentin expression with no E-cadherin expression in all conditions in both immunostaining and western blots. Both lines showed fibronectin expression under each condition, though bands for BxPC-3 were very faint. Compared to cells cultured on 2D, Oligomer appeared to promote fibronectin expression in BxPC-3 and both fibronectin and vimentin expression in MiaPaCa-2 (Fig 8E). It is noteworthy that this upregulation was observed even though GAPDH (loading control) for 2D samples appeared heavier, despite efforts to load equal protein amounts for all samples. The heavier banding for 2D samples is likely due to some residual matrix protein in 3D samples, which would effectively decrease the ratio of cellular protein to total protein for 3D samples compared to 2D samples.

**Fig 9:**
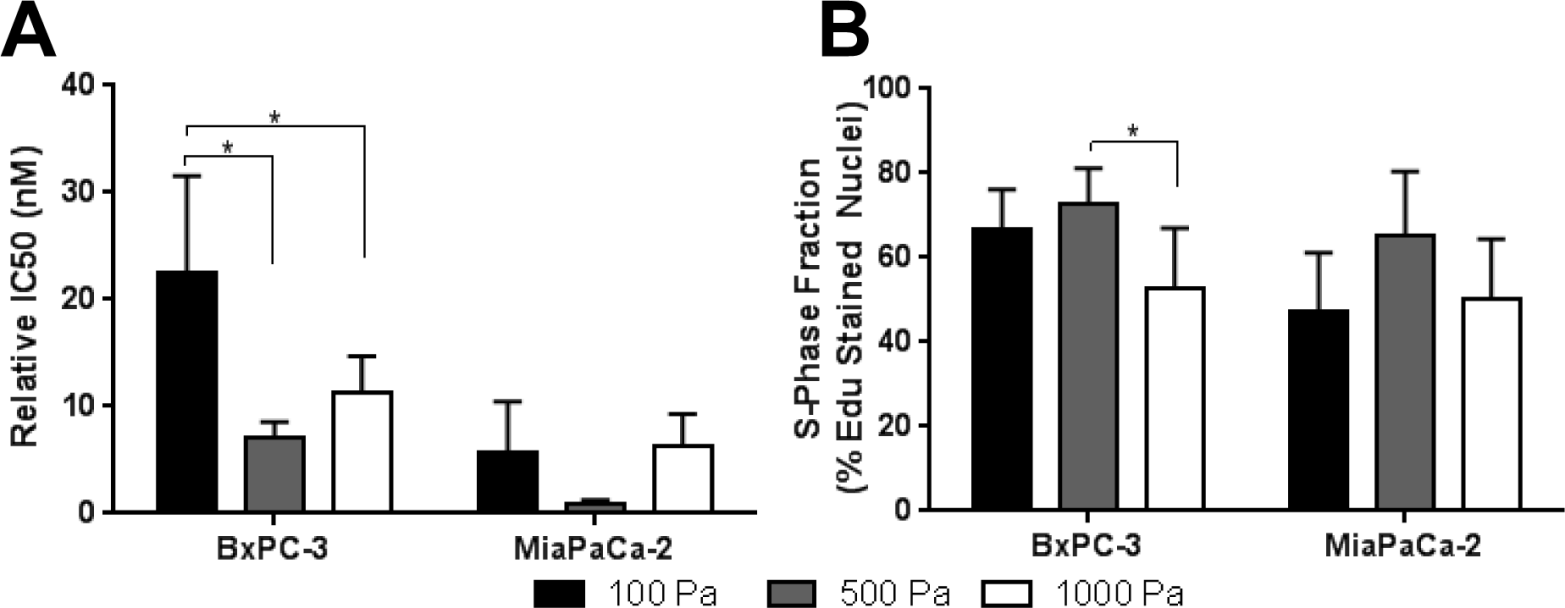
Stromal interstitial matrix stiffness alters PDAC cell gemcitabine sensitivity and proliferative capacity. (A) Gemcitabine IC50 values (mean ± SD; n = 3) and (B) S-phase fraction (mean ± SD; N = 2, n = 3) for BxPC-3 and MiaPaCa-2 cells cultured for 4 days within 3D Oligomer (2x10^5^ cells/mL) prepared at stiffness values of 100 Pa (0.9 mg/mL), 500 Pa (2 mg/mL), and 1000 Pa (3 mg/mL). Asterisk (*) indicates statistically different groups (p<0.05); note that values between different cell types were not compared.

Finally, gemcitabine sensitivity and S-phase fraction were measured for BxPC-3 and MiaPaCa-2 cells within Oligomer of varied stiffness. Only IC50 values for BxPC-3 in 100 Pa were statistically (p<0.05) different from 500 Pa and 1000 Pa; however, both lines showed apparent inverted bell-shaped relationships with minima at 500 Pa (Fig 9A). Interestingly, these minima in gemcitabine sensitivity appeared to correlate with maxima in S-phase fraction, also at 500 Pa, albeit with no statistical significance (Fig 9B). Overall, these results show that matrix stiffness, altered through changing fibril density, can be a regulating factor in not only EMT, but also cell cycle progression and drug sensitivity.

## Discussion

*In-vitro* tumor models provide important basic research and drug screening tools; however, few adequately recreate metastasis and associated EMT, which remains the major cause of PDAC and other cancer-related deaths. The present work focused on 3D tumor-ECM model development, emphasizing the need for more accurate recapitulation of the PDAC desmoplastic ECM composition and microstructure, which is typically characterized by high levels of fibrillar type I collagen [53]. This fibrous, stiff ECM microenvironment is generally thought to compromise drug transport, decrease tumor chemosensitivity, and enhance EMT and tumor invasion [4,6,54]. However, our 3D tumor-ECM model revealed that PDAC phenotype and behavior vary significantly with initial tumor cell phenotype and depend largely on both the biochemical and biophysical properties of the surrounding stromal ECM. Although fibrillar type I collagen was found to generally promote enhanced expression of specific mesenchymal marker proteins, PDAC morphology as well as the extent of cell-ECM interactions and phenotypic heterogeneity were largely determined by matrix biophysical properties, namely fibril density and associated matrix stiffness. Such results are consistent with recent *in-vivo* preclinical studies that have shown dense type I collagen-fibril architectures restrain rather than promote PDAC EMT and metastasis [55,56].

The PDAC stromal ECM is comprised of BM and IM–two ECM types that differ substantially in their molecular composition, microstructure, and viscoelastic properties [57,58]. BM provides a thin, mechanically weak barrier that separates epithelium (e.g., glands, ducts) and endothelium (e.g., blood vessels, lymph vessels) from underlying interstitial tissues. It is primarily composed of type IV collagen which forms highly intertwined supramolecular networks with laminin, entactin, nidogen, and other molecules [59]. In contrast, IM is primarily composed of type I collagen which exhibits hierarchical self-assembly to form the D-banded fibrillar architecture of interstitial connective tissues. During IM synthesis and fibril self-assembly, intermolecular cross-links form between collagen molecules by an enzyme-mediated process, imparting significant stability and strength to the fibrillar matrix [60]. These two ECM types are of particular interest to PDAC because poor patient prognosis has been correlated with a decrease in BM proteins and a corresponding increase in IM content [53,61].

In the present work, Matrigel was used to approximate the BM, and Oligomer was used to recreate and tune the fibrillar IM microenvironment. While no research tools exist today that allow accurate BM recreation *in vitro*, Matrigel, a murine tumor BM extract, has been used extensively to mimic the BM in 3D cancer models. Although Matrigel as a natural hydrogel formulation contains many BM components, it does not exhibit the molecular cross-linking or architecture found in BM *in vivo* and shows significant lot-to-lot variability and low mechanical properties [57,62]. Oligomer, on the other hand, is a soluble type I collagen subdomain that, unlike conventional monomer formulations (atelocollagen and telocollagen), retains mature intermolecular cross-links formed *in vivo* [25]. As a result, this formulation exhibits high-order supramolecular assembly, giving rise to highly branched fibril networks with significantly improved physiological relevance, mechanical integrity, and resistance to proteolytic degradation [23,24,32,63]. Because Oligomer is standardized based upon its self-assembly or fibril-forming capacity, it supports tunability over a broad range of fibril microstructures and matrix stiffness values and shows excellent reproducibility within and between laboratories [23,32]. The distinct self-assembly capacity of Oligomer has been documented in previous published work, where polymerization of Oligomer over the concentration range of 0.5–4 mg/mL yields matrices with G’ values of 40–1500 Pa while matrices prepared with telocollagen and atelocollagen, formulations commonly used in other studies, cover G’ ranges of 2-300Pa and 2-50Pa, respectively [23]. The use of Oligomer in the present work allowed PDAC cells to engage type I collagen IM in a natural fibrillar context with systematic control of fibril density and associated matrix stiffness.

**Fig 10:**
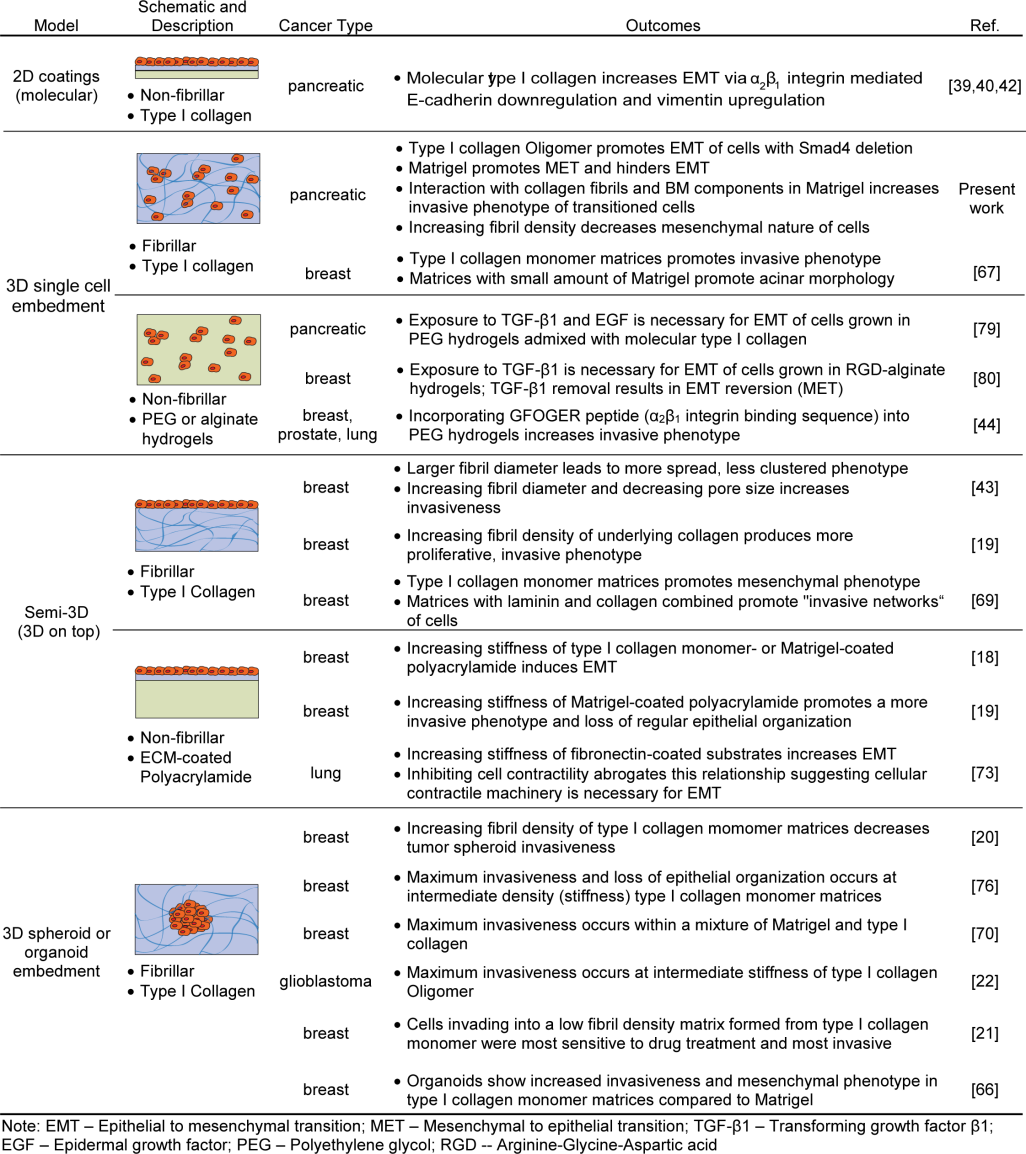
Models used to study role of ECM on EMT and invasion.

Fig 10 summarizes various *in-vitro* tumor models used for EMT and invasion studies, highlighting culture format, ECM scaffold material, and ECM biophysical properties as critical design parameters for recreating pathophysiologically relevant cell-matrix interactions *in vitro* [17,64]. Based on this review, the majority of EMT and invasion studies performed to date have focused on breast cancer, underscoring a need for more pancreatic cancer investigations. Most work targeting ECM-guided EMT has been performed using cell monolayers grown on either 2D plastic surfaces with adsorbed matrix proteins or on-top of 3D substrates (also known as semi-3D). On the other hand, studies focused on tumor invasion, a hallmark of metastasis, involve embedding multi-cellular spheroids or organoids within 3D matrices prepared from monomeric type I collagen. Although not addressed specifically in the table, this review also revealed that culture format often dictates whether cellular or population level analyses are used and that 3D embedment experiments often lack population level evaluations [65–67]. Finally, only one other tumor EMT study was identified that involved 3D embedment of single cells within a fibrillar type I collagen matrix, which is more representative of the tumor IM than Matrigel or bioengineered hydrogels, such as those prepared from polyethylene glycol (PEG) or alginate. These gaps are addressed, in part, by the present work.

Since modulation of the tumor ECM context and associated EMT are likely more of a continuum rather than a binary change [68], the influence of ECM composition and microstructure on tumor cell phenotypic transition was documented by varying IM:BM (Oligomer: Matrigel) ratio while maintaining a constant matrix stiffness. Interestingly, when three different PDAC lines were exposed separately to low-density Oligomer (100 Pa), they maintained their mesenchymal phenotype or showed evidence of EMT, with resultant morphology and protein expression profiles dependent upon initial cell line phenotype. On the other hand, Matrigel (100 Pa) inhibited EMT, promoted a clustered morphology for all cell types, and decreased mesenchymal protein expression. Such observations are consistent with the general expectation that IM is associated with mesenchymal behavior, and BM is involved in maintaining epithelial phenotype [66,67,69]. However, few *in-vitro* studies have demonstrated how simultaneous interaction of tumor cells with IM and BM contributes to EMT and phenotypic heterogeneity. Those studies that have done so, did not examine matrix microstructure and mechanics or compare different cell lines across the EMT spectrum [69,67,70].

In the present work, Matrigel induced epithelial BxPC-3 cells to grow as tight clusters, even when it was present in small amounts together with fibrillar type I collagen. Only in the 100:0 ratio, which had no Matrigel, did a subset of BxPC-3 undergo EMT, as indicated by a subpopulation of cells demonstrating spindle-shaped morphology, prominent vimentin staining, and some loss of E-cadherin staining. Population analysis by western blots supported the notion of matrix-induced EMT heterogeneity with little change in vimentin or E-cadherin expression and faint upregulation of fibronectin, another common marker for the mesenchymal phenotype. These findings for the epithelial PDAC line are consistent with a recent study where embedded normal mammary epithelial cells, MCF-10, only appeared invasive in monomeric type I collagen matrices [67]. When even small amounts of Matrigel were added to type I collagen matrices, MCF-10 displayed a more epithelial acinar phenotype.

On the other side of the EMT spectrum, MiaPaCa-2, which are classified as mesenchymal, showed more drastic morphological changes, as well as a decrease in mesenchymal protein expression as IM:BM ratio increased. An interesting observation was that combined IM and BM interactions in the 75:25 ratio appeared to promote a more invasive, elongated morphology than IM alone. Benton and colleagues reported similar findings with mesenchymal breast cancer cells, MDA-MB-231, which displayed the most invasive, migratory phenotypes when cultured on-top or embedded as spheroids within matrices prepared from monomeric type I collagen and Matrigel [69,70]. Although the above-mentioned studies did not evaluate the matrix physical properties of IM-BM combinations, we noted that the 75:25 ratio was the only mixture of IM and BM that maintained the branched fibrillar architecture of type I collagen and the associated matrix stiffness (100 Pa). In summary, these observations suggest that for epithelial cells, Matrigel promotes epithelial morphogenesis regardless of the presence of an interconnected collagen fibrillar matrix, but for mesenchymal tumor cells, a stable, interconnected collagen fibrillar matrix with sufficient mechanical integrity is a primary factor in driving invasive phenotypes. These results also point to the need for further work to define how complex cell-ECM interactions such as those found at the tumor-stroma interface contribute to phenotypic heterogeneity and the continuum of EMT phenotypes observed *in vivo* [71].

Comparisons between our results and those in Fig 10 also revealed that the manner in which cells sense and respond to changes in substrate stiffness is highly dependent on the *in-vitro* model format (e.g., coated 2D, semi-3D, 3D embedded). Our results showed promotion of EMT and mesenchymal characteristics (e.g. spindle-shaped morphologies, vimentin and fibronectin expression) in PDAC cells embedded within relatively low-density collagen fibril matrices formed from Oligomer. When fibril density was increased, the resulting increased matrix stiffness and spatial constraints hindered mesenchymal-like cell spreading and resulted in confined, clustered growth. These results contradict observations from semi-3D models which have shown cells on-top of soft substrates maintained epithelial characteristics (clustered growth, E-cadherin expression, BM deposition) while increased substrate stiffness increased EMT-like behaviors including cell spreading, migration, and vimentin or fibronectin expression [18,19,72,73]. This trend was observed whether the underlying substrate on which cells were cultured was fibrillar type I collagen or non-fibrillar coatings of molecular type I collagen, Matrigel, or fibronectin on-top of PA gels. Therefore, the observed discrepancy between our results and those of semi-3D models is likely due to differences in geometric constraints. Specifically, the forced apical-basal polarization of cells imposed by semi-3D model geometry has been shown to alter the composition of cell-matrix adhesions and downstream signaling pathways in a manner different than 3D embedment models [74,75]. Assuming that EMT correlates with invasiveness, our results align more closely with those from 3D spheroid embedment models in which the most invasive phenotypes were observed in either the lowest or an intermediate stiffness type I collagen matrix while remaining more clustered at higher stiffness [20–22,76]. These 3D embedment models, including the present work, demonstrate that physical constraints experienced by cells within dense 3D matrices dominate over any increased signaling that may occur from increased ligand density or increased substrate stiffness as observed with semi-3D models. While some *in-vitro* studies aim to decouple matrix density and stiffness through non-natural matrix crosslinking [67,77], the relevance of these methods remains unclear since these two variables are naturally coupled with desmoplasia *in vivo* [56,77]. Taken together, these comparisons highlight that when developing accurate *in-vitro* tumor-ECM models, it is not just the presence of specific ECM ligands or “going 3D” that is important, but one must consider culture geometry, ligand presentation, and fibrillar architecture since all these features dictate EMT mechanobiology *in vivo*.

Our results also highlight two important, often overlooked aspects of 3D *in-vitro* models–the cell-matrix tension balance and the collagen fibril architecture [74,78]. In cancer, loss of cell-matrix tensional homeostasis and cell-induced alignment of type I collagen fibrils have been implicated in tumor progression and invasion [2,19]. Specifically, researchers have shown that increasing matrix tension by applying passive strain to monomeric type I collagen matrices increased invasiveness of embedded mammary organoids [76]. On the other hand, relieving intra-matrix tension by detaching similar collagen matrices from a culture dish caused mammary epithelial cells to grow as tight acinar-like structures rather than the invasive, spindle-shaped morphologies observed in attached matrices [67]. In the present work, the high levels of Matrigel in low IM:BM ratios (50:50, 25:75) disrupted collagen fibril interconnectivity, which likely hindered cells ability to generate force and create tensional strain within the matrix. These mechanical changes, along with the BM signaling from Matrigel, are likely what caused clustered cell growth of both cell lines in these matrices. On the other hand, the type I collagen composition and interconnected fibril matrix of Oligomer (100:0) were sufficient to induce EMT of BxPC-3. Contrary to this result, non-fibrillar hydrogels such as collagen-PEG or RGD-alginate have been shown to not promote EMT in embedded pancreatic or breast cancer cells unless exogenous growth factors, such as TGF-β1, were added [79,80]. It is important to note here that bioengineered materials such as those fashioned from alginate or PEG have no inherent bioactivity and lack physiologically relevant architecture. Even when molecular collagen (non-fibrillar) or RGD ligands are added to promote cell adhesion the architecture, mechanical properties, and resultant cell-substrate interactions that occur within these artificial microenvironments are dramatically different from those experienced by cells *in vivo*. From these observations, it is clear that in-vitro models of tumor EMT and invasion should be designed to accurately recreate ECM architectural features and cell-matrix mechanics for improved correlation between *in-vitro* and *in-vivo* behavior [2,74].

In addition to highlighting the importance of collagen fibril architecture in mechanistic study of tumor EMT and mechanobiology, observations from this work demonstrate the translational potential of Oligomer-based tumor-ECM models. Oligomer is unique among type I collagen formulations because of its purity, standardization, and ability for user customization, making it a powerful tool for creating standardized 3D models [23,25,30]. Additionally, its molecular make-up and its ability to form tissue-like collagen matrices with mature intermolecular crosslinks make Oligomer an ideal material for predicting *in-vivo* outcomes through recreating the ECM environment of desmoplastic tumors such as PDAC which are rich in type I collagen [3,24,25,53]. In fact, the observed trend in increased EMT behavior with decreased matrix density in the present work, has been recently noted in genetically engineered mouse models of PDAC [55,56]. In these studies, reduced stromal cell activity led to decreased desmoplasia and tumor stiffness which led to increased EMT, invasion and metastasis, as well as lower overall survival rates. The matrix-induced EMT heterogeneity in low-density Oligomer matrices is also reminiscent of observations from human clinical tumor samples in which only a subpopulation of cells undergoes EMT [71,81]. Finally, although only minor significant differences in drug sensitivity were found, this study demonstrates our ability to perform drug dosing experiments and generate IC50 values for these 3D tumor-ECM models. Collectively, these observations showcase the potential of Oligomer to serve as a robust platform for mechanistic study of metastasis and creation of predictive 3D drug screening models.

## Conclusion

This work serves as a first step in the development of novel *in-vitro* 3D tumor-ECM models where Oligomer is used as a standardized type I collagen formulation to recreate and customize the IM component. From the foundational understanding of PDAC desmoplasia and EMT gained from these experiments, we can now develop more complex models of pancreatic and other cancers to systematically define the role of other prominent components of the tumor stromal microenvironment and study tumor invasion in more detail. Additionally, the model of matrix-driven EMT created by embedding PDAC cells within Oligomer-based fibril matrices provides a useful tool that can be applied to further mechanistic study. Finally, by developing and applying standardized *in-vitro* models with defined ECM microenvironments, we are moving closer to accurately recreating tumor-stroma interactions and desmoplasia to provide pathophysiologically relevant PDAC models which can be used for phenotypic drug screening to ultimately predict therapeutic response and improve patient outcomes.

## Supporting information

S1 Table Comparison of gemcitabine IC50 values for PDAC lines cultured on 2D plastic. (DOCX)
