# Supplementary material for: 3D collagen fibrillar microstructure guides pancreatic cancer cell phenotype and serves as a critical design parameter for phenotypic models of EMT
Source: PLoS One. 2017 Nov 30;12(11):e0188870. doi: 10.1371/journal.pone.0188870 (PMC5708668; doi:10.1371/journal.pone.0188870)
Supplement: S1 Table — (DOCX) [file pone.0188870.s001.docx]

**S1 Table. Comparison of gemcitabine IC50 values for PDAC lines cultured on 2D plastic**

| IC50 (nM) | | |  | Experimental Conditions | | | | Reference |
| --- | --- | --- | --- | --- | --- | --- | --- | --- |
| BxPC-3 | Panc-1 | MiaPaCa-2 |  | Cell Density  (10^3^ cells/well) | Culture Medium | Duration  (hr) | Assay |  |
| 830 | 95 | 494 |  | 4 | RPMI for all | 72 | WST-1 | [1] |
| 13.6 | 137 | 7.73 |  | 2 | RPMI for BxPC-3; DMEM for Panc-1 & MiaPaCa-2 | 96 | Cell Titer Glo | [2] |
| 5,910 | 58,200 | 11,430 |  | 5 | RPMI for all but MiaPaCa-2 | 72 | MTT | [3] |
| 17.9 | 27 | 51 |  | 20 | DMEM for all | 72 | PI intensity | [4] |
| 25,300 | 30,700 | 25,900 |  | 1-5 | RPMI for BxPC-3; DMEM for Panc-1 & MiaPaCa-2 | 72 | MTT | [5] |
| 80 | 225 | 733 |  | 4 | RPMI for all but Panc-1 | 48 | PI intensity | [6] |
| 12.6 | 28.2 | 1.9 |  | 4 | RPMI for BxPC-3; DMEM for Panc-1 & MiaPaCa-2 | 72 | Alamar Blue | Present study |

Note: Grayed rows represent studies with experimental conditions and results most similar to the present study (also grayed)

**References for S1 Table**

[1] N. Awasthi, C. Zhang, A.M. Schwarz, S. Hinz, C. Wang, N.S. Williams, M. a. Schwarz, R.E. Schwarz, Comparative benefits of nab-paclitaxel over gemcitabine or polysorbate-based docetaxel in experimental pancreatic cancer, Carcinogenesis. 34 (2013) 2361–2369.

[2] G. Hu, F. Li, K. Ouyang, F. Xie, X. Tang, K. Wang, S. Han, Z. Jiang, M. Zhu, D. Wen, X. Qin, L. Zhang, Intrinsic gemcitabine resistance in a novel pancreatic cancer cell line is associated with cancer stem cell-like phenotype, Int. J. Oncol. 40 (2012) 798–806.

[3] W. Huanwen, L. Zhiyong, S. Xiaohua, R. Xinyu, W. Kai, L. Tonghua, Intrinsic chemoresistance to gemcitabine is associated with constitutive and laminin-induced phosphorylation of FAK in pancreatic cancer cell lines, Mol. Cancer. 8 (2009).

[4] N. Kurata, H. Fujita, K. Ohuchida, K. Mizumoto, P. Mahawithitwong, H. Sakai, M. Onimaru, T. Manabe, T. Ohtsuka, M. Tanaka, Predicting the chemosensitivity of pancreatic cancer cells by quantifying the expression levels of genes associated with the metabolism of gemcitabine and 5-fluorouracil, Int. J. Oncol. 39 (2011) 473–482.

[5] S. Mori-Iwamoto, Y. Kuramitsu, S. Ryozawa, K. Taba, M. Fujimoto, K. Okita, K. Nakamura, I. Sakaida, A proteomic profiling of gemcitabine resistance in pancreatic cancer cell lines, Mol. Med. Rep. 1 (2008) 429–434.

[6] M.J. Rathos, K. Joshi, H. Khanwalkar, S.M. Manohar, K.S. Joshi, Molecular evidence for increased antitumor activity of gemcitabine in combination with a cyclin-dependent kinase inhibitor, P276-00 in pancreatic cancers, J. Transl. Med. 10 (2012) 161.
